# Online Resources for Hidradenitis Suppurativa for Patient Use: Systematic Search and Analysis

**DOI:** 10.2196/72773

**Published:** 2025-08-22

**Authors:** Emily Sheetz, Aryn A Alanizi, Joshua Edwards, Alice A Roberts

**Affiliations:** 1 Eastern Virginia Medical School, 721 Fairfax Ave, Norfolk, VA, 23507, United States, 1 6106099276

**Keywords:** hidradenitis suppurativa, online resources, patient education

## Abstract

This research letter evaluates the quality and readability of hidradenitis suppurativa (HS) websites found on Google and Bing with the DISCERN instrument and Flesch-Kincaid Readability metrics. Comprehensive and reliable articles can lead to increased knowledge about HS and further enhance physician-patient relationships and shared decision-making. This study’s aim was to identify reliable resources to help bridge knowledge gaps and support informed discussions on management and treatment options.

## Introduction

Hidradenitis suppurativa (HS) is a chronic, inflammatory skin condition that is often challenging to diagnose, with delays averaging 7 to 10 years [1]. Its complex clinical course and psychosocial burden lead many patients to the internet for information and treatment options. While online resources can support shared decision-making and physician-patient communication [2], the accuracy and readability of content can vary widely. High-quality, accessible information empowers patients, but misleading or difficult-to-comprehend information causes confusion and hinders effective management. We assessed the quality and readability of HS-related websites using the DISCERN instrument and Flesch-Kincaid metrics.

## Methods

A systematic search of Google and Bing was conducted using the term *hidradenitis suppurativa* in an incognito browser with location services disabled. Searches were performed on the same day to minimize discrepancies due to search algorithm changes. Twenty results were obtained from each search engine. Advertisements, duplicate content, paywalled articles, and incomplete sources were screened out, leaving a combined 20 websites for analysis.

Two independent reviewers used the DISCERN instrument to evaluate health information based on 16 questions covering clarity, references, and treatment (Multimedia Appendix 1) [3]. DISCERN uses a 5-point scale, with higher scores indicating better quality. Scores from both reviewers were averaged. Readability was measured using the Flesch-Kincaid Grade Level, which determines the US school grade level required for comprehension [4]. *P* values were calculated using independent 2-tailed *t* tests to compare DISCERN scores, while readability metrics were summarized descriptively.

## Results

The mean DISCERN scores for Google (Alphabet) and Bing (Microsoft) were 54.05 (SD 11.53) and 59.83 (SD 9.73), respectively, indicating good quality [5]. Websites authored or reviewed by physicians had significantly higher DISCERN scores (62.1 vs 49.7; *P*=.02) than those by nonphysicians, indicating that expert involvement improves the quality of online health content. However, the mean reading grade levels for Google (10.8, SD 2.4) and Bing (10.5, SD 1.9) exceeded the National Institutes of Health recommendation for a sixth or seventh grade level [6]. Only half of physician-reviewed articles met this criterion. Table 1 summarizes our findings, highlighting a significant gap between content quality and accessibility, underscoring the need for improved patient-friendly resources.

Moreover, websites found on Bing exhibited statistically significant differences in DISCERN question 7 (providing additional sources of support*; P*=.03) and a trend, although nonsignificant, in question 10 (treatment benefit descriptions*; P*=.06).

**Table 1.** Hidradenitis suppurative websites analyzed.

Website	Search engine	Author or reviewer	Mean DISCERN score	Reading grade level
National Health Service	Google	Academic institution	52.5	9.6
Mayo Clinic	Google and Bing	Academic institution	67.5	9.7
American Academy of Dermatology	Google and Bing	Physician	70	6.3
National Institutes of Health	Google	Physician	63.5	11.5
MedlinePlus	Google and Bing	Academic institution	42.5	8.1
DermNet	Google and Bing	Physician	56	12.6
Cleveland Clinic	Google and Bing	Academic institution	61	10.3
HS Foundation	Google and Bing	Nonprofit organization	47	10.4
Medscape	Google	Physician	62.5	11
WebMD	Google	Physician	70	5.7
Nationwide Children’s Hospital	Google	Hospital	37.5	7.4
Wikipedia	Google and Bing	Global site	61	9.7
Mount Sinai	Google	Academic institution	33	11.1
British Skin Foundation	Google and Bing	Charity	49	9.6
American Osteopathic College of Dermatology	Google	Academic institution	46	11.1
Cedars-Sinai	Google	Physician	47	6.4
Patient.Info	Bing	Physician	65.5	8.4
Healthline	Bing	Physician	71.5	7.6
FamilyDoctor.org	Bing	Physician	50	7.5
Medical News Today	Bing	Physician	65	9.4

## Discussion

While the internet is a valuable resource for patient education, many HS-related websites may be difficult for patients to understand. Given the chronic and distressing nature of HS, access to clear and reliable information is of utmost importance. The variability in readability and quality indicates a need for greater oversight and standardization in online medical content. Complex resources may discourage patients with lower health literacy from engaging with important health information, leading to misinformation, delays in seeking professional care, and suboptimal self-management strategies [7]. High readability demands on websites may further widen health disparities, as HS is more prevalent among individuals with lower socioeconomic status, who may also have lower health literacy. To improve equitable access to medical information, resources should be written in plain language, include visual aids, and be available in multiple languages to accommodate diverse patient backgrounds.

Health care providers should also guide patients to reliable sites, ideally incorporating links to after-visit summaries or patient portals. Future efforts should focus on improving the clarity of online HS resources without compromising their informational value. Website developers could also involve patients in the content creation process to ensure materials are accessible.

Despite these findings, several limitations must be considered. First, the study focused on the top search results for Google and Bing at a specific time point, which may not reflect dynamic changes in search algorithms. Notably, Bing yielded more support group links, potentially due to algorithm prioritization; Google often ranks academic sources higher, while Bing surfaces more user-friendly content. Additionally, the readability analysis relied on established formulas, which primarily assess sentence length and word complexity. These tools do not account for contextual factors such as formatting choices or visual aids, which may improve comprehension. Finally, although patient forums like Reddit may offer valuable insights, they are not professionally curated and should be interpreted with caution.

In conclusion, we found considerable variability in the quality and readability of online HS resources. Due to the persistent and often debilitating course of HS, trustworthy and comprehensible resources are crucial to support understanding and management of this condition. As online health information continues to shape patient perceptions and behaviors, improving the quality and readability of digital medical content should remain a priority.

## Supplementary material

10.2196/72773 Multimedia Appendix 1 DISCERN questionnaire.

## Abbreviations

HS: hidradenitis suppurativa
