# Supplementary material for: Online Resources for Hidradenitis Suppurativa for Patient Use: Systematic Search and Analysis
Source: JMIR Dermatol. 2025 Aug 22;8:e72773. doi: 10.2196/72773 (PMC12373301; doi:10.2196/72773)
Supplement: Multimedia Appendix 1 [file derma-v8-e72773-s001.docx]

Appendix 1:

| **Section 1: Is the publication reliable?** | |
| --- | --- |
| Question 1 | Are the aims clear? |
| Question 2 | Does it achieve its aim? |
| Question 3 | Is it relevant? |
| Question 4 | Is it clear what sources of information were used to compile the publication (other than the author or producer)? |
| Question 5 | Is it clear when the information used or reported in the publication was produced? |
| Question 6 | Is it balanced and unbiased? |
| Question 7 | Does it provide details of additional sources of support and information? |
| Question 8 | Does it refer to areas of uncertainty? |
| **Section 2: How good is the quality of information on treatment choices?** | |
| Question 9 | Does it describe how each treatment works? |
| Question 10 | Does it describe the benefits of each treatment? |
| Question 11 | Does it describe the risks of each treatment? |
| Question 12 | Does it describe what would happen if no treatment is used? |
| Question 13 | Does it describe how the treatment choices affect overall quality of life? |
| Question 14 | Is it clear that there may be more than one possible treatment choice? |
| Question 15 | Does it provide support for shared decision making? |
| **Section 3: Overall rating of the publication** | |
| Question 16 | Based on the answers to all of the above questions, rate the overall quality of the publication as a source of information about treatment choices. |
